# Sirtuin 1 rs1467568 and rs7895833 in South African Indians with early-onset coronary artery disease

**DOI:** 10.5830/CVJA-2015-085

**Published:** 2016

**Authors:** Prithiksha Ramkaran, Devapregasan Moodley, Anil A Chuturgoon, Alisa Phulukdaree, Sajidah Khan

**Affiliations:** Discipline of Medical Biochemistry and Chemical Pathology, University of KwaZulu-Natal, Durban, South Africa; Discipline of Medical Biochemistry and Chemical Pathology, University of KwaZulu-Natal, Durban, South Africa; Discipline of Medical Biochemistry and Chemical Pathology, University of KwaZulu-Natal, Durban, South Africa; Department of Physiology, School of Medicine, Faculty of Health Sciences, University of Pretoria, South Africa; Department of Cardiology, Nelson R Mandela School of Medicine, University of KwaZulu-Natal, Durban, South Africa

**Keywords:** sirtuin 1, rs1467568, rs7895833, single-nucleotide polymorphism, premature coronary artery disease, South African Indians

## Abstract

**Background:**

Sirtuin 1 (SIRT1), a class III histone deacetylase, has been identified as a candidate molecule affecting the epigenetic mechanisms of cardiovascular disease (CVD). Previous studies have shown that some SIRT1 single-nucleotide polymorphisms (SNPs) are associated with body mass index, diabetes, blood pressure, cholesterol metabolism and coronary artery calcification. We investigated two A>G SIRT1 SNPs, rs1467568 and rs7895833, in young South African (SA) Indians with coronary artery disease (CAD) and compared them to Indian and black controls.

**Methods:**

For rs1467568, a total of 287 subjects were recruited into this study (104 CAD patients, 99 age-, gender- and race-matched controls, and 84 age- and gender-matched black controls). For rs7895833, a total of 281 subjects were recruited into this study (100 CAD patients, 99 age-, gender- and race-matched controls, and 82 age- and gender-matched black controls). All patients were male, of Indian ethnicity, stable CAD confirmed on angiography, mean age 37.5 years; range 24–45. All subjects were genotyped using TaqMan SNP genotyping assays.

**Results:**

The variant allele for both SNPs was found at a higher frequency in the total Indian group compared to the total black population (rs1467568: 41 vs 18.5%, respectively, p < 0.0001, OR = 3.190, 95% CI: 2.058–40943; and rs7895833: 41 vs 22%, respectively, p < 0.0001, OR = 2.466, 95% CI: 1.620– 3.755). Indian controls presented with a higher frequency for both SNPs compared to black controls (rs1467568: 40 vs 18.5%, respectively, p < 0.0001, OR = 2.996, 95% CI: 1.850– 4.853; and rs7895833: 41 vs 22%, respectively, p < 0.0001, OR = 2.513, 95% CI: 1.578–4.004). No difference was seen in the distribution of both SNPs between CAD patients and either control group. We did not observe any association between the SNPs and clinical parameters in CAD patients and controls.

**Conclusion:**

Both SNP variant alleles occurred more frequently in SA Indians than in SA blacks. A larger study group and further analysis is required to assess whether these SIRT1 SNPs may serve as risk factors that contribute to Indians developing early-onset CAD.

## Background

Sirtuins are a class of NAD+-dependent proteins involved in a wide range of biological processes such as aging, transcription, apoptosis and inflammation.^1^ Sirtuin 1 (SIRT1) is located in the nucleus and cytoplasm, and plays an important role in epigenetic regulation by deacetylating a range of transcription factors to control downstream gene expression.^2^ The targets of SIRT1 include Forkhead box O (FOXO)1, (FOXO)3, peroxisome proliferator-activated receptor gamma coactivator 1-alpha (PGC-1α), tumour suppressor p53, nuclear factorkappa B (NF-κB), Notch, hypoxia-inducible factor (HIF) 1α, liver X receptor (LXR), farnesoid X receptor (FXR) and sterol regulatory element-binding protein (SREBP)1c.^3^

Recent studies have demonstrated a protective role of SIRT1 in atherosclerosis, the underlying process of coronary artery disease (CAD).^4^ SIRT1 performs an anti-inflammatory function by downregulating the expression of several pro-inflammatory cytokines by interfering with the NF-κB signalling pathway. By deacetylating NF-κB, SIRT1 suppresses the expression of lectin-like oxidised low-density lipoprotein receptor-1 (Lox- 1), a scavenger receptor for oxidised low-density lipoproteins (oxLDL), therefore preventing foam cell formation.^4^ SIRT1 controls the activity of LXR, an important regulator of lipid homeostasis and inflammation.^4^ Activation of LXR results in expression of ATP-binding cassette (ABC) transporter ABCA1, which regulates the removal of cholesterol into high-density lipoproteins (HDL), a process known as reverse cholesterol transport (RCT). Dysfunctional RCT could lead to accumulation of cholesterol, thus stimulating foam cell production and the progression of atherosclerosis.^4^,^5^ Given the important role of SIRT1 in cardiovascular disease, research on genetic variation in the SIRT1 gene has become of interest.

Genetic variations such as single-nucleotide polymorphisms (SNPs) in the SIRT1 gene have been associated with inflammation, body mass index, type 2 diabetes, blood pressure and dyslipidaemia, all of which are well-established risk factors for CAD.^2^,^6^-^19^ Coronary artery disease remains a leading cause of mortality worldwide, with an unusually high prevalence of earlyonset disease among the Indian population. South African (SA) Indians have a much higher prevalence of CAD compared to SA blacks.^10^ There are currently no studies on SIRT1 SNPs in SA Indians with CAD. We therefore investigated the SIRT1 A>G SNPs, *rs1467568* and *rs7895833* in young SA Indians with CAD and compared them to Indian and black controls.

## Methods

A total of 287 subjects were recruited into the *SIRT1 rs1467568* study (104 CAD patients, 99 age-, gender- and race-matched controls, and 84 age- and gender-matched black controls) following institutional ethical approval (BE067/14). The inclusion criteria for CAD patients were: Indian ancestry and unrelated adult males aged < 45 years, and stable CAD confirmed on angiography. The exclusion criteria for controls included an acute coronary syndrome/revascularisation procedure in the preceding three months, chronic renal or liver disease, malignancy and known inflammatory or infectious disease.

Blood samples were obtained following an overnight fast. A full pathology report of clinical markers was assessed by routine laboratory testing at the Global Clinical and Viral Laboratory (Durban, South Africa), a South African national accreditation system (SANAS) certified laboratory. The following parameters were tested: haematology (Roche Sysmex 1800XT), chemistry (Beckman Coulter DXC600), endocrinology and high-sensitivity C-reactive protein (hsCRP) (Siemens Centaur XP) and serology (BD Biosciences FACS Calibur), as per international standards to obtain levels of total cholesterol, HDL-C, LDL-C, triglycerides, fasting glucose, two-hour glucose, fasting insulin, glycosylated haemoglobin, sodium, potassium, bicarbonate, chloride, urea, creatinine, glomerular filtration rate, CD4, CD8, CD45 and CD3 count. The physical measurements of weight, height, abdominal circumference, waist circumference and patient history were conducted by the cardiologist (Dr S Khan).

Genomic DNA was extracted from the whole blood sample of each patient and control, according to the method described by Sambrook *et al.*^11^ Cells were transferred to 600-μl lysis buffer [0.5 % sodium dodecyl sulphate (SDS), 150 mM NaCl, 10 mM ethylenediaminetetra-acetic acid (EDTA), 10 mM Tris–HCl (pH 8.0)]. To this, RNase A (100 μg/ml; DNase-free) was added to the solution and incubated at 37°C for one hour. Proteinase K (200 μg/ml) was then added and incubated for three hours at 50°C 

Protein contaminants were then precipitated by adding 5 mM 0.1% potassium acetate before centrifugation at 5 000 × g for 15 min. Supernatants containing genomic DNA were transferred to fresh tubes and extracted with 100% isopropanol on ice, and thereafter washed with 70% ethanol. DNA samples were dissolved in 10 mM Tris and 0.1 mM EDTA (pH 7.4, 4°C). DNA concentration was determined using the Nanodrop 2000 spectrophotometer, and all samples were standardised to a concentration of 10 ng/μl.

Following the manufacturer’s protocol, TaqMan® SNP predesigned genotyping assay (Life Technologies, Cat #4351379) was used to genotype all subjects for both SNPs. The TaqMan® genotyping assay contains two primers for amplifying the sequence of interest and two TaqMan® minor groove-binding (MGB) probes for detecting alleles. The presence of two probe pairs in each reaction allows genotyping of the two possible variant alleles at the SNP site in a DNA target sequence.

The genotyping assay determines the presence or absence of a SNP based on the change in fluorescence of the dyes associated with the probes. The TaqMan® MGB probes consist of targetspecific oligonucleotides with a reporter dye at the 5′ end of each probe: one VIC®-labelled probe to detect allele 1 sequence (A-allele in the case of *rs1467568* and *rs7895833*) and one FAM™-labelled probe to detect allele 2 sequence (G-allele in the case of *rs1467568* and *rs7895833*). A fluorescence signal for both dyes indicates heterozygous for allele 1–allele 2 (AG).

A final reaction mixture consisted of 40 × TaqMan® predesigned genotyping assay, 2 × TaqMan® genotyping master mix, nuclease-free water, and a 10-ng genomic DNA template. The experiment was done using the Applied Biosystems® ViiA™ 7 Real-Time PCR system.

## Statistical analysis

The Hardy–Weinberg equilibrium was used to test for deviation of allele/genotype frequency. All other statistical analyses were performed with Graphpad prism software (version 5.0). Allele and genotype frequencies were calculated using the Fisher’s exact and chi-squared tests, respectively. The comparison of biochemical measures between the wild type and variant genotypes was done with a non-parametric t-test. Results are expressed as mean ± standard error. A p-value less than 0.05 was considered statistically significant.

## Results

## SIRT1 rs1467568

The genotype distribution complied with the Hardy–Weinberg equilibrium in the CAD patients and Indian controls (chi-squared p = 0.233 and p = 0.941, respectively), but not in the black control group (chi-squared p < 0.05).

No significant difference was observed in the distribution of the *SIRT1 rs1467568* alleles between the CAD patients and Indian controls (41 vs 40% respectively, p = 0.9196, OR = 1.040, 95% CI: 0.6998–1.545). The Indian controls presented with a higher frequency of the variant allele compared to the black controls (40 vs 18.5%, respectively, p < 0.0001, OR = 2.996, 95% CI: 1.850–4.853). The variant allele was found at a higher frequency in the total Indian group compared to the total black population (41 vs 18.5%, respectively, p < 0.0001, OR = 3.057, 95% CI: 1.974–4.733) (Table 1).

**Table 1:** *SIRT1 rs1467568* and *rs7895833* genotype and allele frequencies in CAD patients and controls)

**	*CAD patients *	*SA Indian controls*	*Total SA Indians *	*SA black controls *
*SIRT1 rs1467568*	(n = 104) n, (%)	(n = 99) n, (%)	(n = 203) n, (%)	(n = 84) n, (%)
Genotypes				
AA	40 (38.46)	36 (36.36)	76 (37)	62 (73.81)
AG	42 (40.38)	46 (46.46)	88 (43)	13 (15.48)
GG	22 (21.15)	17 (17.17)	39 (19)	9 (10.71)
Alleles				
A	122 (59)	118 (60)	240 (59)	137 (81.5)
G	86 (41)	80 (40)	166 (41)	31 (18.5)
*SIRT1 rs7895833*	(n = 100)	(n = 99)	(n = 199)	(n = 82)
Genotypes				
AA	36 (36)	34 (34.34)	70 (35)	47 (57.32)
AG	47 (47)	48 (48.48)	95 (48)	34 (41.46)
GG	17 (17)	17 (17.17)	34 (17)	1 (1.22)
Alleles				
A	119 (59.5)	116 (59)	235 (59)	128 (78)
G	81 (40.5)	82 (41)	163 (41)	36 (22)

## SIRT1 rs7895833

The genotype distribution complied with the Hardy–Weinberg equilibrium in the CAD patients, Indian controls and black controls (chi-squared p = 0.970, p = 1.000 and p = 0.164, respectively).

No significant difference was observed in the distribution of the *SIRT1 rs7895833* alleles between CAD patients and Indian controls (40.5 vs 41%, respectively, p = 0.9188, OR = 0.9629, 95% CI: 0.6457–1.436). The Indian controls presented with a higher frequency of the variant allele compared to the black controls (41 vs 22% respectively, p < 0.0001, OR = 2.513, 95% CI: 1.578– 4.004). The variant allele was found at a higher frequency in the total Indian group compared to the total black population (41 vs 22% respectively, p < 0.0001, OR = 2.466, 95% CI: 1.620–3.755) (Table 1).

Phulukdaree and co-workers reported biochemical measures of CAD patients and healthy controls in 2012.^12^ As expected, in our study, CAD patients presented with more conventional risk factors, such as higher body mass index (BMI), higher total and LDL cholesterol and triglyceride levels, and a higher prevalence of type 2 diabetes mellitus than the control groups. No association between the SIRT1 SNPs and biochemical measures were found in the CAD patients (Table 2), Indian controls (Table 3) and black controls (Table 4).

**Table 2:** Characteristics of CAD patients according to the SIRT1 rs1467568 and SIRT1 rs7895833 genotypes

	SIRT1 rs1467568 genotype			SIRT1 rs7895833 genotype		
	*Wild type (AA)*	*Variant (AG+GG)*	*p-value*	*Wild type (AA)*	*Variant (AG+GG)*	*p-value*
BMI (kg/m^2^)	27.52 ± 0.81	28.57 ± 0.55	ns	28.02 ± 0.80	28.33 ± 0.59	ns
Total cholesterol (mmol/l)	5.73 ± 0.32	5.17 ± 0.20	ns	5.32 ± 0.24	5.46 ± 0.25	ns
LDL (mmol/l)	3.70 ± 0.29	3.27 ± 0.21	ns	3.41 ± 0.23	3.47 ± 0.24	ns
HDL (mmol/l)	0.98 ± 0.04	0.89 ± 0.03	ns	0.91 ± 0.04	0.93 ± 0.04	ns
Triglycerides (mmol/l)	2.41 ± 0.28	2.37 ± 0.18	ns	2.34 ± 0.24	2.38 ± 0.20	ns
Fasting glucose (mmol/l)	6.48 ± 0.50	6.27 ± 0.33	ns	6.18 ± 0.47	6.32 ± 0.34	ns
Fasting insulin (μlU/ml)	16.97 ± 2.21	15.54 ± 1.12	ns	14.17 ± 1.19	16.95 ± 1.61	ns
HBA_1c_ (%)	6.63 ± 0.33	6.61 ± 0.24	ns	6.57 ± 0.34	6.60 ± 0.24	ns
hsCRP (mg/l)	9.83 ± 2.58	6.97 ± 0.98	ns	8.93 ± 2.42	7.78 ± 1.31	ns
IL-6 (pg/ml)	2.80 ± 0.90	2.45 ± 0.59	ns	2.41 ± 0.80	2.73 ± 0.68	ns

BMI = body mass index, LDL = low-density lipoprotein, HDL = high-density lipoprotein, HBA_1c_ = glycated haemoglobin, hsCRP = high-sensitivity C-reactive protein, IL-6 = interleukin-6, ns = non-significant.

**Table 3:** Characteristics of Indian controls according to the SIRT1 rs1467568 and SIRT1 rs7895833 genotype

	SIRT1 rs1467568 genotype			SIRT1 rs7895833 genotype		
	*Wild type (AA)*	*Variant (AG+GG)*	*p-value*	*Wild type (AA)*	*Variant (AG+GG)*	*p-value*
BMI (kg/m^2^)	25.88 ± 0.93	26.65 ± 0.69	ns	25.14 ± 1.01	26.88 ± 0.66	ns
Total cholesterol (mmol/l)	5.32 ± 0.16	5.54 ± 0.13	ns	5.56 ± 0.19	5.41 ± 0.12	ns
LDL (mmol/l)	3.47 ± 0.13	3.86 ± 0.12	ns	3.88 ± 0.17	3.63 ± 0.11	ns
HDL (mmol/l)	1.04 ± 0.07	0.91 ± 0.03	ns	0.97 ± 0.07	0.95 ± 0.03	ns
Triglycerides (mmol/l)	1.79 ± 0.22	1.92 ± 0.27	ns	1.63 ± 0.17	2.00 ± 0.27	ns
Fasting glucose (mmol/l)	5.59 ± 0.34	5.38 ± 0.16	ns	5.27 ± 0.20	5.56 ± 0.22	ns
Fasting insulin (μlU/ml)	15.91 ± 1.96	16.72 ± 1.66	ns	13.36 ± 1.46	18.03 ± 1.75	ns
HBA_1c_ (%)	5.78 ± 0.21	5.65 ± 0.11	ns	5.85 ± 0.16	5.63 ± 0.13	ns
hsCRP (mg/l)	4.58 ± 0.60	7.95 ± 1.71	ns	6.52 ± 1.41	6.87 ± 1.57	ns
IL-6 (pg/ml)	2.16 ± 0.79	2.86 ± 0.63	ns	2.83 ± 0.87	2.48 ± 0.60	ns

BMI = body mass index, LDL = low-density lipoprotein, HDL = high-density lipoprotein, HBA_1c_ = glycated haemoglobin, hsCRP = high-sensitivity C-reactive protein, IL-6 = interleukin-6, ns = non-significant.

**Table 4:** Characteristics of black controls according to the SIRT1 rs1467568 and SIRT1 rs7895833 genotype

	SIRT1 rs1467568 genotype			SIRT1 rs7895833 genotype		
	*Wild type (AA)*	*Variant (AG+GG)*	*p-value*	*Wild type (AA)*	*Variant (AG+GG)*	*p-value*
BMI (kg/m^2^)	25.53 ± 0.54	27.13 ± 1.20	ns	25.58 ± 0.63	26.57 ± 0.87	ns
Total cholesterol (mmol/l)	4.12 ± 0.12	4.47 ± 0.23	ns	4.30 ± 0.13	4.14 ± 0.18	ns
LDL (mmol/l)	2.62 ± 0.10	3.02 ± 0.22	ns	2.78 ± 0.12	2.71 ± 0.16	ns
HDL (mmol/l)	1.05 ± 0.045	1.03 ± 0.088	ns	1.08 ± 0.05	0.99 ± 0.06	ns
Triglycerides (mmol/l)	0.99 ± 0.072	0.93 ± 0.15	ns	0.98 ± 0.08	0.96 ± 0.11	ns
Fasting glucose (mmol/l)	4.80 ± 0.084	4.90 ± 0.11	ns	4.87 ± 0.11	4.77 ± 0.08	ns
Fasting insulin (μlU/ml)	7.69 ± 0.67	12.11 ± 3.74	ns	9.21 ± 1.73	8.59 ± 1.10	ns
HBA_1c_ (%)	5.83 ± 0.062	5.79 ± 0.082	ns	5.87 ± 0.07	5.76 ± 0.077	ns
hsCRP (mg/l)	6.64 ± 1.86	5.82 ± 1.23	ns	7.81 ± 2.42	4.83 ± 0.84	ns

BMI = body mass index, LDL = low-density lipoprotein, HDL = high-density lipoprotein, HBA_1c_ = glycated haemoglobin, hsCRP = high-sensitivity C-reactive protein, IL-6 = interleukin-6, ns = non-significant.

## Discussion

Indian populations throughout the world show early-onset CAD, one to two decades earlier than other ethnic groups.^13^ South African Indians have the highest mortality rates due to CAD, while black South Africans have a very low prevalence of the disease.^10^

Increasing evidence has shown that SIRT1 is involved in CAD by regulating a number of key metabolic and physiological processes. SIRT1 serves as an anti-atherosclerotic factor by mediating endothelial nitric oxide synthase (eNOS) and improving endothelial dysfunction, regulating inflammation, reversing cholesterol transport and reducing the risk of CAD. 14

Several SNPs have been identified in SIRT1, a candidate molecule involved in the epigenetic regulation of CAD. To date, there are only a few human genetic association studies regarding SIRT1 SNPs and CAD. Our study was the first investigation of *SIRT1 rs1467568* and *rs7895833* in SA Indian CAD patients. We observed that the variant alleles of both SIRT1 SNPs occurred more frequently in SA Indians compared to SA blacks. We did not observe any difference in allele frequencies between CAD patients and control groups.

Previous studies have shown that some of the SIRT1 SNPs are associated with BMI and obesity, glucose tolerance and diabetes, blood pressure, cholesterol metabolism and coronary artery calcification, all of which contribute to the CAD phenotype.^15^-^19^ We examined the possible association between rs1467568 and rs7895833 in SIRT1 and BMI, and levels of total cholesterol, LDL, HDL, triglycerides, fasting glucose, fasting insulin, HbA_1c_, hsCRP, or IL-6 in CAD patients and control groups, but did not observe any association.

The Rotterdam study investigated SIRT1 variation (assessed by three tagging SIRT1 SNPs: *rs7895833, rs1467568* and *rs497849*) in relation to BMI and risk of obesity in 4 573 participants, including 413 individuals with prevalent and 378 with incident type 2 diabetes mellitus (T2DM).^20^ In homozygous carriers with prevalent T2DM, the SIRT1 haplotype 1 had 1.9 times (95% CI: 1.1–3.2) increased risk of CVD mortality compared to non-carriers.

An intended replication study (Erasmus Rucphen family study) was carried out involving 2 347 participants. Both studies observed that the minor alleles of rs7895833 (G allele) and rs1467568 (A allele) were associated with lower BMI and a 13–18% decreased risk of obesity in two independent Dutch populations.^17^ In another study, the A allele of rs7895833 was associated with increased risk of obesity and hypertension in Japanese men.^15^

Recent studies investigated the association between SIRT1 SNPs (*rs7895833, rs7069102, rs144124002* and *rs2273773*) and CAD in a Turkish population. While *rs7069102, rs2273773* and *rs144124002* were significantly associated with increased risk for CAD, they found no association between *rs7895833* and CAD.^21^,^22^

Shimoyama *et al*. reported that SIRT1 *rs7069102* and *rs2273773* were associated with abnormal cholesterol metabolism and coronary artery calcification, respectively, in Japanese haemodialysis (HD) patients. The study also found that the A allele frequency of SIRT1 *rs7895833* and G allele frequency of *rs7069102* were significantly lower in HD patients compared to controls, suggesting an impact on survival.^19^

The allele frequencies of *rs7895833* and *rs1467568* show ethnic variation, and this is a possible reason for differing disease patterns among populations. The frequency of the *rs7895833* A allele was relatively low (0.29) in Japanese compared to Dutch, Turkish and Caucasian subjects who had similar allele frequencies (0.80, 0.85 and 0.80, respectively).^15^,^17^,^23^ The A allele of *rs1467568* (reported as the protective allele) showed marked difference in frequency between European (0.25) and Japanese (0.84) subjects.^23^

## Conclusion

Both SNP variant alleles occurred more frequently in SA Indians than in SA blacks, but no difference was found between CAD patients and controls. This study is limited by sample size and a larger study may be required to fully assess the functional significance of these polymorphisms.
